# Bisphosphonates as anticancer agents in early breast cancer: preclinical and clinical evidence

**DOI:** 10.1186/s13058-015-0634-8

**Published:** 2015-09-02

**Authors:** Daniele Santini, Luciano Stumbo, Chiara Spoto, Loretta D’Onofrio, Francesco Pantano, Michele Iuliani, Marco fioramonti, Alice Zoccoli, Giulia Ribelli, Vladimir Virzì, Bruno Vincenzi, Giuseppe Tonini

**Affiliations:** Department of Medical Oncology, Campus Bio-Medico University of Rome, Via Alvaro del Portillo 200, 00128 Rome, Italy

## Abstract

Bisphosphonates (BPs) are approved as standard therapy in breast cancer for the treatment of bone metastases, since they were demonstrated to reduce the prevalence of skeletal-related events including fractures and hypercalcemia. In the adjuvant setting, BPs can be given to prevent and treat tumor therapy-induced bone loss in premenopausal and postmenopausal women and, owing to their beneficial effect on bone turnover, have also been evaluated for prevention of bone metastases occurrence. In this article we will review the mechanisms through which BPs have been demonstrated to prevent premetastatic niche formation and cell proliferation in bone lesions. Moreover, preclinical evidence of antitumoral effects of BPs will be presented and results from the most important clinical trials will be described critically. BPs may clearly play a clinically important role in early breast cancer in a postmenopausal adjuvant setting.

## Introduction

Bone is a common site for metastases from breast cancer (BC), and 70 % of patients with advanced disease demonstrate bone involvement [1]. Bisphosphonates (BPs) are approved in BC as standard therapy for prevention of skeletal-related events (SRE) of bone metastases. The antiresorptive effects of the nitrogen-containing BPs (N-BPs), including alendronate, risedronate, ibandronate, and zoledronate, appear to result from their inhibition of farnesyl pyrophosphate synthase (FPPS) in osteoclasts. FPPS is a key enzyme in the mevalonate pathway, which generates isoprenoid lipids utilized for the post-translational modification of small guanosine triphosphatases (GTPases) (e.g., Ras, Rho, and Rac). These proteins, in turn, are essential for osteoclast survival and function. In addition, N-BPs have been shown to induce the production of an intracellular adenosine triphosphate analog that can directly induce cellular apoptosis and modulate the immune response [2]. As a result, N-BPs interfere with multiple cellular functions required for the bone-resorbing activity and survival of osteoclasts.

BPs have been demonstrated in advanced settings to reduce the prevalence of SRE, including in pathological fractures, radiotherapy, spinal compression, bone surgery, and hypercalcemia. In the adjuvant setting, BPs can be given to prevent and treat tumor therapy-induced bone loss in premenopausal and postmenopausal women, and owing to their beneficial effect on bone turnover, have also been evaluated for prevention of bone metastases and extraskeletal recurrence in early breast cancer (EBC).

The bone metabolic rate may influence the homing of cancer cells by increasing blood flow. The subsequent cancer cell migration out of the blood vessel and through the tissue requires appropriate signals from chemoattracting molecules (such as CXCL12) released in the extracellular matrix during bone resorption [3]. The metabolic activity of bone is also likely to provide growth factors that have the potential to both enhance survival and promote cancer cell proliferation. Additionally, bone marrow (BM)-derived stem cells are of fundamental importance in the development of metastases at other sites, preparing the environment for tumor cells to establish metastasis [4]. Drugs able to target bone can therefore provide an additional strategy to prevent bone metastasis, expanding the role of BPs in the management of BC [5]. Numerous preclinical experiments have shown that development of bone metastases can be inhibited by BPs through both bone-mediated and direct antitumor mechanisms. The emerging clinical trial results suggest an increasing role for adjuvant BPs in the treatment of EBC, although benefits appear to be confined mostly to the postmenopausal setting.

## Review

### Disseminated tumor cells, premetastatic niche and BPs

Progression of the primary tumor can prepare distant sites known as the premetastatic niche, for the arrival of disseminated tumor cells (DTCs) [6]. In BC, tumor cells have an innate predilection for growth within the bone microenvironment [7] and the dissemination of malignant cells to bone is thought to be an early event. Indeed, the presence of DTCs in BM is a common phenomenon observed in 30–40 % of patients with primary EBC.

Once established in putative metastatic niches in bone, tumor cells can remain in a dormant state for several years under the control of environmental signals, and in many cases never develop into clinically noticeable metastasis. Cancer cells entering the BM need to escape or avoid dormancy and begin proliferating to effectively produce a metastatic tumor [8].

Signals from the bone microenvironment are essential to determine DTC fate, and BM is an ideal “soil” able to promote growth of the primary “seed” [9]. The BM microenvironment indeed contains supportive niches for hematopoiesis and generation of the cells that remodel bone. The interactions between cancer cells, osteoblasts, and osteoclasts, and both hematopoietic and endothelial stem cells, support cancer cell survival and proliferation within the bone microenvironment [10].

The presence of DTCs in the BM predicts the risk of recurrence of cancer in patients with EBC [11]. Significant correlation between DTC persistence in BM and poor prognosis has been demonstrated in several studies [12]. Additionally, it has been shown that DTCs in BC patients are able to survive chemotherapy [13], because BM can provide an ideal sanctuary for these cancer cells to evade systemic anticancer therapy [14]. For these reasons, alternative therapeutic options that improve elimination of DTCs may reduce the risk of relapse and improve survival in patients with EBC.

Although it is not yet clear which specific factors determine the fate of DTCs and facilitate their persistence, it has been postulated that DTCs in the BM can be activated by osteoclast-mediated release of bone-derived growth factors [15]. Elevated levels of bone turnover in the EBC setting have been shown to promote bone metastases development [16]. Using BPs to inhibit bone turnover and to block osteolysis could therefore prevent survival of DTCs before they establish metastasis [17], and recent clinical evidence supports this hypothesis.

In several phase II clinical studies including women with high-risk EBC, both zoledronic acid (ZA) and ibandronate in combination with standard adjuvant therapy were demonstrated to effectively increase DTC clearance and reduce the DTC number and persistence in BM compared with standard therapy alone [18, 19]. In particular, Aft et al. [20] demonstrated that patients with DTC-free BC treated with monthly ZA were more likely to remain DTC-free at 3 months (*P* = 0.03), and that the subset of patients with estrogen receptor (ER)-negative and human epidermal growth factor receptor 2 (HER-2)-negative disease were more likely to have pathologic complete response with ZA versus no ZA. In another study, ZA (4 mg/month) decreased DTC levels by 12 months (*P* <0.0006) and 24 months (*P* = 0.0026) compared to baseline in DTC-positive patients with BC [21]. Banys et al. [22] analyzed the influence of ZA on DTC in BM and survival of EBC patients in a prospective clinical trial. Patients with DTC-positive BM were randomized to treatment with ZA (plus chemotherapy) or placebo (and chemotherapy). All patients treated with ZA became BM-negative after 24 months in comparison with 84 % in the control group (*P* = 0.032). No significant correlation between BP therapy and clinical outcome was observed, but patients presenting with persistent DTCs 12 months after diagnosis had significantly shorter overall survival (OS) (*P* = 0.011).

A recent single-center study showed that BP treatment has no significant influence on disease-free survival (DFS) or OS in DTC-negative patients, but is significantly associated with increased DFS (*P* <0.001) and OS (*P* = 0.006) in DTC-positive patients [23]. Consequently, bone-targeted agents such as BPs appear to have not only a stabilizing effect on the bone density itself, but also to have antitumorigenic activity. Preclinical and clinical evidence has supported this antitumoral effect of BPs in EBC, enriching knowledge of the mechanisms of action in this setting and of interactions with factors such as the hormonal status of patients.

### Preclinical evidence of antitumoral effect of BPs

There is growing evidence of BP anticancer activity in preclinical BC model systems [24]. Several in-vitro and in-vivo studies suggested both direct and indirect effects of BPs on the tumor microenvironment. Indeed, anticancer activity of BPs may result from an indirect effect through the inhibition of bone resorption and consequent reduction in bone-derived growth factors. At the same time, direct antitumor effects, leading to inhibition of tumor cell invasion, adhesion, and proliferation, have also been described in BC preclinical models. Preclinical in vitro studies demonstrate BPs to be internalized into BC cells and to exert direct antitumor effects via inhibition of tumor cell adhesion [25], invasion [26], and proliferation [27], in addition to induction of tumor cell apoptosis [28].

In contrast to in vitro studies, N-BP antitumoral activity in animal models appears mostly to be obtained indirectly rather than via a direct cytotoxic effect [29]. ZA creates a less favorable bone microenvironment for the survival of DTCs via inhibition of osteoclast-mediated bone resorption; for example, ZA deprives tumor cells of bone-derived growth factors (e.g., transforming growth factor beta) that are required for the seeding and growth of tumor cells in the BM [30]. ZA may also exert its effects on the BM microenvironment, on osteoblasts, macrophages, and myeloid-derived suppressor cells, but also on mesenchymal stem cells (MSCs) [31, 32]. Gallo et al. [33] demonstrated in vitro that ZA significantly reduces the migration of MSCs and affects the ability of MSCs to secrete RANTES and interleukin-6, two growth factors promoting BC cell migration. N-BPs may also act indirectly on tumor cells through antiangiogenic and immunomodulatory mechanisms. Wood et al. [34] observed significant antiangiogenic properties of ZA in vitro and in vivo, describing a modulator effect of BPs on endothelial cell migration.

The literature shows some evidence supporting the interesting hypothesis of tumor angiogenesis inhibition by BPs in vivo. Both pamidronate [35] and ZA [36] demonstrated a statistically significant decrease, compared with basal values, in vascular endothelial growth factor levels after a single infusion in cancer patients. However, in contrast to pamidronate, ZA induced a more prolonged decrease in serum vascular endothelial growth factor up to 21 days after the infusion. Moreover, ZA also induced a significant, although transient, decrease in serum platelet-derived growth factor. A subsequent study confirmed these results showing the potential antiangiogenic role of ZA in vivo, with an innovative dose schedule referred to as metronomic for ZA infusion (1 mg every week) instead of the standard regimen (4 mg every 28 days) [37]. Therapeutic doses of BPs have also been shown to modulate monocyte, macrophage, and dendritic cell function with an evident immunomodulatory activity. Moreover, ZA enhances direct natural killer cytotoxicity against tumor cells [38] and stimulates the expansion and the cytotoxicity [39] of Vγ9Vδ2 T cells, a subset of human T cells with antitumor activity. Finally, BPs in BC may prevent tumor cell invasion to bone by enhancing the antitumor effect of antineoplastic agents. Several preclinical studies have shown sequence-dependent synergy between chemotherapy agents (doxorubicin, paclitaxel, oral tegafur-uracil, cyclophosphamide-methotrexate-5-fluorouracil, epirubicin and docetaxel) and ZA [40].

### Clinical evidence of antitumoral effect of BPs

Despite tremendous optimism about adjuvant BP therapy, results from large trials are heterogeneous and sometimes conflicting, as summarized in Table 1. Diel et al. [41] first reported a potential OS benefit of oral clodronate in a prospective clinical trial. These findings were initially confirmed by Powles et al. [42] in 1,089 patients under adjuvant treatment for BC, and were subsequently contradicted by Saarto et al. [43]. Inconclusive results were also reported from the randomized, double-blind, placebo-controlled NSAPB B-34 trial involving 3,323 patients with EBC, in whom oral clodronate given for 3 years did not improve DFS and OS versus placebo in the overall population [44]. Important information was collected when results from studies evaluating zoledronate as a bone-protective agent in patients with endocrine treatment were presented. For postmenopausal patients, the Z-FAST and ZO-FAST trials investigated the efficacy of immediate versus delayed zoledronate (4 mg intravenously every 6 months for 5 years) to prevent therapy-related bone loss in patients with hormone receptor-positive stage I–III BC, who were receiving endocrine therapy (letrozole) [45]. Assessing DFS at 60 months as a secondary endpoint, a 34 % reduction in relative risk was demonstrated in the ZO-FAST trial [46].

**Table 1:** Major trials exploring bisphosphonate activity in early breast cancer

Trial	Number of patients	Regimen	Duration (years)	Combination therapy	DFS benefit (+/−)	Bone loss benefit (+/−)
Z-Fast [45]	600	Immediate versus delayed ZA	5	Letrozol	NR	+
ZO-FAST [46]	1,065	Immediate versus delayed ZA	5	Letrozol	+	+
NSAPB-34 [44]	3,323	Oral clodronate	3	Standard therapy according to stage	– (population as a whole)	+
AZURE [47]	3,360: premenopausal, 1,504; postmenopausal, 1,531; unknown, 324	ZA	5	Standard therapy according to stage	– (but significant improvements in DFS in postmenopausal women)	+
Saarto et al. [43]	299	Oral clodronate	3	Standard therapy according to stage	29	NR
NATAN [48]	693	ZA	5	Standard therapy according to stage	– (trend for women aged >55)	NR

*DFS* disease-free survival, *NR* not reported, *ZA* zoledronic acid

The AZURE trial was the first study to explore the antitumor activity of zoledronate combined with (neo)adjuvant chemotherapy [47]. A total of 3,360 patients were randomly assigned to receive standard adjuvant systemic therapy either with or without zoledronate. Trial results suggest no overall benefit from the addition of ZA to standard adjuvant treatments for EBC, but demonstrate significant reduction in the development of bone metastases in women with established menopause. Starting from these findings the role of zoledronate in a postneoadjuvant setting was studied in the NATAN trial [48], where patients were randomized to ZA for 5 years versus no postoperative treatment if they did not reach complete response. There was a trend toward longer DFS only in patients aged >55 years, and no benefit on clinical outcome in the general population. At the San Antonio Breast Cancer Symposium 2013, Coleman et al. [49] presented a large meta-analysis collecting more than 22,982 patients from 36 trials. Also in that work the ability of BPs to reduce distant recurrence (predominantly in bone) appears to be largely confined to postmenopausal women (distant recurrence: 18.4 % in women on BPs vs 21.9 % in women not on BPs, *P* = 0.0003; bone recurrence: 5.9 % and 8.8 %, respectively, *P* <0.00001). Interestingly, reductions in bone recurrence and BC deaths occurred regardless of ER status, nodal status, type of BP, and whether or not women received chemotherapy. The impact on survival and fracture rates for the use of ZA versus no use (or delayed use) in the adjuvant treatment of patients with EBC was also evaluated in the meta-analysis by Valachis et al. [50]. This work provided evidence in favor of ZA use in the adjuvant BC setting in terms of survival benefit. Findings from ABCSG-12 in premenopausal women, ZO-FAST in postmenopausal women, and AZURE in both premenopausal and postmenopausal women suggest that hormonal effects on the bone microenvironment may play a substantial role in determining who may benefit most from adjuvant ZA therapy.

Adjuvant BPs have shown highly promising results in postmenopausal women receiving letrozole for EBC [43] and in premenopausal women receiving endocrine therapy [51], but no significant improvements in DFS or OS across unselected populations of patients with EBC [41, 47]. However, a consistent improvement in both DFS and OS with administration of adjuvant BPs has emerged post menopause, either natural [41, 43, 49] or induced by goserelin [48].

A meta-analysis including six trials, together specifically evaluating effects of adjuvant BPs on DFS according to menopausal status, reported no beneficial effect in the entire population of patients with EBC treated by BPs compared with the control arm [52]. A significant DFS benefit was reported only in the subgroup of women with established menopause (hazard ratio (HR) = 0.81 (95 % confidence interval (CI): 0.69–0.95)), while a potential detrimental role of adjuvant BPs was observed in premenopausal and perimenopausal women. This latter observation was made in the AZURE trial, which highlighted a significant detrimental effect of ZA on the rate of non-skeletal metastases in premenopausal women, independent of the ER status of the tumor (HR = 1.32 (95 % CI: 1.09–1.59)] [44]. Saarto et al. [43] came to a similar conclusion in a study of clodronate.

Bone has long been identified as a special metastatic microenvironment and its cells are acutely sensitive to changes in endocrine status. The increase in osteoclastic bone resorption occurring in menopause, caused by estrogen deficiency and ovarian failure, makes premenopausal and postmenopausal bone niches different as host environments for disseminated cancer cells.

In the preclinical setting, Ottewell et al. [53] recently showed that ovariectomy (OVX) increases bone resorption and induces growth of disseminated tumor cells in bone, without effects in other sites. Tumors were detected in 83 % of animals after OVX (postmenopausal model) compared with 17 % after a sham operation (premenopausal model) [53]. ZA inhibited OVX-induced bone tumor growth but had no effect in sham-operated animals, supporting the observed benefit of antiresorptive therapy in patients with postmenopausal BC and the lack of benefit of adjuvant ZA in premenopausal BC patients. Current clinical data suggest that both hormone suppression and reduction of bone turnover-derived growth factors are needed for sufficient suppression of dormant micrometastases in patients with EBC. Furthermore, the role of BM-derived stem cells in the development of extraskeletal metastases might be influenced by the patient's endocrine status, and BPs may help to maintain BM-derived stem cells and cancer cell dormancy in BM in the absence of an estrogen microenvironment. The AZURE trial supports this supposition in a subset analysis demonstrating that the potential anticancer activity of ZA observed in postmenopausal women occurs outside bone [44]. It has been hypothesized that an increase in the rate of relapse would be associated with increased bone resorption, which creates a bone microenvironment potentially serving as a homing site for DTCs [16]. In the clinical setting, the MA27 study recently supported this notion indirectly with some speculation resulting from comparison of anastrazole and exemestane in postmenopausal women with EBC. The study reported no difference between the two aromatase inhibitors (AIs) in terms of DFS [54]. However, in a subsequent exploratory analysis, the authors showed that patients who had osteoporosis (self-reporting) and were not receiving therapy for the condition had the highest rate of relapse, compared with those who never had osteoporosis or those who received osteoporosis therapy [55]. This strongly supports the hypothesis that an impaired bone microenvironment related to drug-induced postmenopausal estrogen depletion would provide fertile soil for DTCs, and that osteoporosis (as a surrogate marker of estrogen depletion) would negatively affect the treatment outcomes in EBC patients, which can be significantly reversed by bone antiresorption therapy.

The biological rationale for the benefit of BPs only for postmenopausal patients lies in the estrogen-deficient BM microenvironment, which may lead to bone loss by increased osteoclast activity. Estrogen and BPs may interact at the level of BM cancer cell dormancy. On the other hand, the estrogen-rich bone microenvironment appears to promote the survival and expansion of DTCs in the endosteal niche, because estrogen increases the number and activity of endosteal osteoblasts, which are critical mediators of stem cell dormancy and survival [56]. This observation may suggest that high levels of estrogens in premenopausal women neutralize the capability of BPs to decrease DTCs. As a consequence, a decreased level of estrogens obtained through luteinizing hormone-releasing hormone agonist-mediated ovarian suppression in the premenopausal setting may increase the power of BPs in stopping DTC proliferation.

Recently, the final analysis after median follow up of 94.4 months in the ABCSG-12 trial showed that in premenopausal women with ER-positive EBC receiving adjuvant hormonal treatment with goserelin plus tamoxifen or anastrozole, both with or without ZA (4 mg every 6 months) for 3 years, ZA still reduced the relative risk of disease progression (HR = 0.77; 95 % CI: 0.60–0.99; *P* = 0.042) and risk of death (HR = 0.66; 95 % CI: 0.43–1.02; *P* = 0.064). Absolute risk reductions with ZA were 3.4 % for DFS and 2.2 % for OS. These results suggest that ZA given every 6 months in combination with luteinizing hormone-releasing hormone agonist enhances the efficacy of adjuvant endocrine treatment, and this benefit is maintained long term [57]. In the AZURE trial [47], no DFS and OS improvement was apparent in a premenopausal population. This primary result appears to differ markedly from the findings of Gnant et al. [57] in the ABCSG-12 study, but from an endocrine perspective the postmenopausal patients in the AZURE trial, who had undergone menopause more than 5 years before study entry, were similar to the goserelin-treated patients in the ABCSG-12 study, who had low levels of reproductive hormones at study entry. The explanation for this finding seems unclear, but perhaps bone provides a sanctuary for cancer cells after more than 5 years of menopause when the low levels of estrogens cause an increase in the activity of osteoclasts (i.e., bone resorption). This increase in activity creates a bone microenvironment suitable for receiving the neoplastic cells in the niche. BPs, inhibiting bone resorption, would therefore be able to reduce the formation of the preneoplastic niche and the dissemination of cancer cells to other body sites.

## Conclusions

Even if considered historically only in metastatic bone disease, BPs have emerged as an interesting treatment option for bone loss prevention (and perhaps disease recurrence reduction) in patients with EBC. The ability of BPs to reduce the occurrence of distant metastases appears largely confined to postmenopausal women. Considering the available data, we would recommend the use of BPs in all ER-positive premenopausal women whose treatment regimen includes luteinizing hormone-releasing hormone agonist as supported by preclinical [56] and clinical [57] evidence, or those developing complete ovarian suppression after adjuvant chemotherapy in order to preserve their bone health, and in all patients with EBC in adjuvant treatment with AIs. A field of research is to explore the potential of ZA for interrupting the crosstalk between DTCs and the estrogen-poor bone microenvironment, a step that has been reported to potentially improve DFS in EBC. Meta-analyses did not demonstrate any important difference in disease outcome by type of BP (amino vs non-amino) and we also do not know for how long BP treatment should be administered, and whether this should be for 3 years or longer (Table 2). When considered appropriate, however, adjuvant BPs - either clodronate (1,600 mg/day) or ZA (4 mg every 6 months) -should be recommended to be continued for a minimum of 3 years. Now is the right time to consider BPs for prevention of metastases in the postmenopausal setting of EBC patients.

**Table 2:** Open issues for bisphosphonate adjuvant treatment in early breast cancer trials

Patient-related issues	Biological issues
Histological type (ductal, lobular, etc.)	Preclinical models: how closely do animal models of a low-estrogen environment mimic the postmenopausal status in women with breast cancer?
Molecular subtype (luminal, triple negative breast cancers, etc.)	Type of bisphosphonate (amino vs non-amino): superiority of one bisphosphonate in selective setting?
Clinical and pathological stage of disease	Interaction with hormone therapy and chemotherapy: synergy or not?
Estrogen and progesterone levels	Duration of bisphosphonate therapy: how long?
Menopausal status (different menopausal definition in trials)	
Comorbidities	
Personal factors	

## Abbreviations

AI: aromatase inhibitor
BC: breast cancer
BM: bone marrow
BP: bisphosphonate
CI: confidence interval
DFS: disease-free survival
DTC: disseminated tumor cell
EBC: early breast cancer
ER: estrogen receptor
FPPS: farnesyl pyrophosphate synthase
HR: hazard ratio
MSC: mesenchymal stem cell
N-BP: nitrogen-containing bisphosphonate
OS: overall survival
OVX: ovariectomy
SRE: skeletal-related events
ZA: zoledronic acid
